# Interpretable machine learning for predicting placental abruption in early-onset preeclampsia: model development and evaluation

**DOI:** 10.3389/fmed.2025.1731693

**Published:** 2026-01-12

**Authors:** Lijun Su, Jingli Zhang, Haiying Wu

**Affiliations:** Department of Obstetrics, Henan Provincial People’s Hospital (Zhengzhou University People’s Hospital), Zhengzhou, China

**Keywords:** early-onset preeclampsia, interpretable machine learning, placental abruption, random forest, SHapley Additive exPlanations

## Abstract

**Objective:**

Early-onset preeclampsia (EOPE) represents a particularly severe clinical subtype of preeclampsia (PE) and is frequently complicated by placental abruption, which can result in serious maternal and fetal morbidity or mortality. This study aimed to develop and validate an interpretable machine learning (IML) model for predicting placental abruption in patients with EOPE.

**Methods:**

A retrospective cohort of 580 EOPE patients who delivered between January 2021 and June 2025 was analyzed and randomly divided into training (70%) and validation (30%) sets. Dual-step feature selection combining LASSO regression and the Boruta algorithm identified the most relevant predictors. Six supervised algorithms, including decision tree (DT), k-nearest neighbor (KNN), logistic regression, random forest (RF), support vector machine (SVM), and extreme gradient boosting (XGBoost), were developed and compared. Model performance was evaluated using AUC, F1 score, calibration curve, and decision curve analysis (DCA). SHapley Additive exPlanations (SHAP) were employed for model interpretation.

**Results:**

Eight optimal predictors were selected: urinary protein, placental growth factor (PlGF), diastolic blood pressure (DBP), age, fibrinogen (FIB), prepregnancy BMI, disease severity, and smoking during pregnancy. The RF model achieved the best performance (training AUC = 0.968; validation AUC = 0.894), along with the highest accuracy and F1 score among all algorithms. Calibration curves showed strong consistency between predicted and observed probabilities, and DCA confirmed greater net clinical benefit across a wide range of threshold probabilities. The confusion matrix demonstrated high sensitivity and specificity, indicating stable classification performance. SHAP analysis revealed that urinary protein, PlGF, FIB, and DBP were the dominant predictors, where elevated urinary protein and DBP and reduced FIB and PlGF significantly increased abruption risk.

**Conclusion:**

The SHAP-based RF model demonstrated high predictive accuracy and interpretability, providing a transparent, data-driven framework for individualized risk assessment of placental abruption in EOPE. This interpretable approach may facilitate early risk identification and personalized management in clinical practice.

## 1 Introduction

Preeclampsia (PE) is a pregnancy-specific hypertensive disorder characterized by new-onset hypertension and multi-organ dysfunction after 20 weeks of gestation (1). It affects approximately 3–5% of all pregnancies and remains one of the major causes of maternal and perinatal morbidity and mortality worldwide (2). Among the clinical subtypes of PE, early-onset preeclampsia (EOPE), defined as onset before 34 weeks of gestation, represents a more severe phenotype associated with impaired placentation, pronounced systemic endothelial dysfunction, and adverse maternal-fetal outcomes (3). EOPE is often linked to placental hypoperfusion, oxidative stress, and inflammatory activation, which together create a pathological milieu that predisposes patients to acute obstetric complications (4). One of the most devastating complications of EOPE is placental abruption—the premature detachment of the placenta from the uterine wall before delivery. Placental abruption is associated with catastrophic maternal outcomes such as hemorrhagic shock and disseminated intravascular coagulation, as well as severe fetal hypoxia and death (5–7). The shared etiopathogenesis between EOPE and placental abruption, characterized by abnormal spiral artery remodeling, endothelial injury, and coagulation imbalance, suggests that patients with EOPE are at particularly high risk of developing placental abruption (3, 8, 9). Although several regression-based models have been proposed for predicting preeclampsia-related complications, including placental abruption, their performance remains limited (10, 11). Logistic regression relies on linear assumptions and is unable to model nonlinear or higher-order interactions among angiogenic factors, hemodynamic indices, and maternal characteristics—interactions that are central to EOPE pathophysiology. Recent studies have shown that such models often yield only moderate discrimination and are not routinely used in clinical practice, largely because their functional form restricts their ability to capture the complex multivariable relationships underlying placental disease. These constraints provide a strong rationale for evaluating machine learning (ML) approaches that can flexibly model nonlinear, multivariate interactions (12, 13).

In recent years, ML techniques have emerged as powerful tools for risk prediction and decision support in obstetric medicine (14, 15). ML algorithms are capable of learning complex relationships among high-dimensional clinical, biochemical, and imaging data, enabling early identification of patients at risk for adverse outcomes. Nevertheless, the “black-box” nature of many ML models has hindered their adoption in clinical practice, as clinicians often require not only high predictive accuracy but also transparent reasoning that aligns with pathophysiological understanding (16, 17). To address this limitation, interpretable machine learning (IML) approaches have been developed to balance predictive performance with model transparency. IML integrates feature importance visualization, local explanation methods (e.g., SHapley Additive exPlanations, SHAP), and inherently interpretable algorithms, allowing users to trace how each predictor contributes to individual predictions (18, 19).

Although ML models have been applied to predict the onset of preeclampsia and other pregnancy complications, we found no published studies that developed an interpretable ML model specifically for predicting placental abruption in EOPE. Existing work predominantly focuses on logistic-regression-based models or non-interpretable ML approaches, without incorporating SHAP-based interpretability or dual-step feature selection (20, 21). Therefore, our study addresses an unmet methodological gap by providing the first interpretable ML framework tailored to EOPE-associated placental abruption. By combining robust feature selection with interpretable algorithms, we sought to establish a clinically applicable and explainable tool to assist in individualized risk assessment, early intervention, and optimized perinatal management.

## 2 Materials and methods

### 2.1 Study subjects

**Figure 1:**
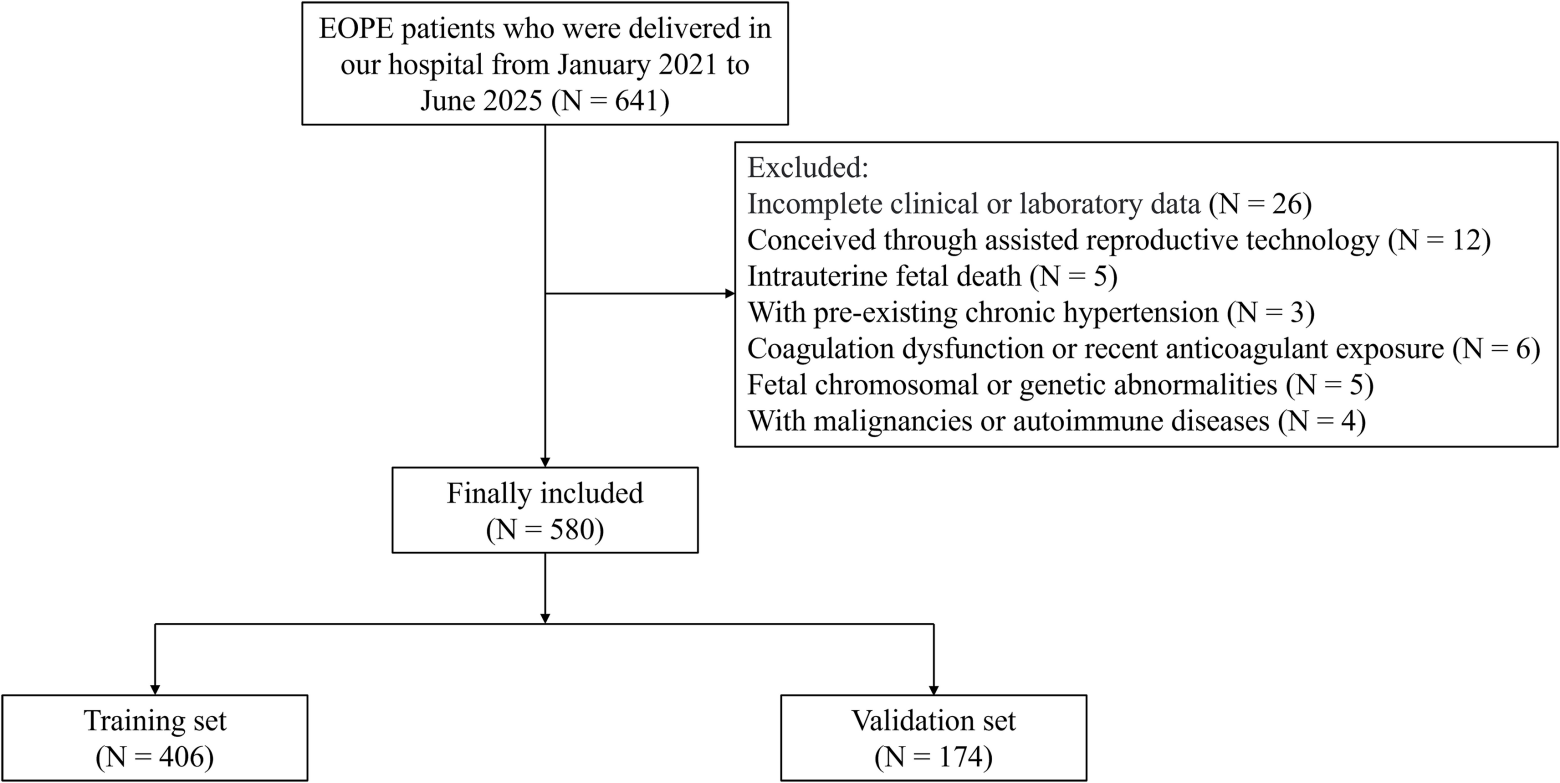
Research flowchart: patient screening, enrollment, and grouping. EOPE, early-onset preeclampsia.

This retrospective study included women diagnosed with EOPE who delivered at our hospital between January 2021 and June 2025. After screening for eligibility and data completeness, a total of 580 cases were identified through electronic medical record review. To construct and validate the predictive model, participants were randomly assigned to a training set (70%) and a validation set (30%) through computer-generated random allocation to minimize selection bias. Inclusion Criteria: (1) Diagnosis of EOPE established before 34 weeks of gestation, based on the American College of Obstetricians and Gynecologists criteria (22); (2) Singleton pregnancy with complete prenatal and delivery records. Exclusion Criteria: (1) With incomplete clinical or laboratory data; (2) Patients who conceived through assisted reproductive technology; (3) Intrauterine fetal death; (4) With pre-existing chronic hypertension; (5) With coagulation dysfunction or recent anticoagulant exposure; (6) Fetal chromosomal or genetic abnormalities; (7) With malignancies or autoimmune diseases (Figure 1).

### 2.2 Diagnostic criteria for EOPE and placental abruption

EOPE refers to cases in which the clinical manifestations of PE appear before 34 weeks of gestation. Diagnosis requires the new onset of hypertension in a previously normotensive woman, defined as systolic blood pressure (SBP) ≥ 140 mmHg and/or diastolic blood pressure (DBP) ≥ 90 mmHg on at least two measurements taken 4 h apart after 20 weeks of gestation. The diagnosis is further supported by the presence of proteinuria [≥0.3 g/24 h, protein/creatinine ratio ≥0.3, or random urine dipstick ≥ (+)]. In situations where proteinuria is absent, PE may still be established if hypertension is accompanied by evidence of maternal organ dysfunction, including low platelet count, elevated liver enzymes, renal impairment, pulmonary congestion, or neurological and visual disturbances (22).

Placental abruption is diagnosed when a normally implanted placenta separates from the uterine wall before fetal delivery, leading to maternal and/or fetal compromise. The diagnosis is primarily clinical but can be supported by imaging and postpartum findings. Typical features include: (i) Abrupt, persistent abdominal or lower back pain, often accompanied by uterine rigidity or tenderness. Vaginal bleeding may be present but is not mandatory, as bleeding can be concealed within the uterine cavity. (ii) Sonographic indicators may reveal a hypoechoic or heterogeneous region between the placenta and myometrium, thickened placental tissue, or localized placental elevation consistent with retroplacental hemorrhage. (iii) Gross placental inspection after delivery may demonstrate dark clots adherent to the maternal surface or depression of the placental tissue corresponding to prior hemorrhage sites. A clinical diagnosis is typically established when criterion (i) coexists with either imaging evidence (ii) or postpartum confirmation (iii) (23).

### 2.3 Data collection

The data included maternal demographic, obstetric, laboratory, and neonatal factors. Maternal demographic variables were age, prepregnancy body mass index (BMI), postpregnancy BMI, and smoking during pregnancy. Obstetric characteristics included SBP and DBP, which were measured during the last hospitalization before delivery, as well as PE severity, gestational age at delivery, number of pregnancies (gravidity), mode of delivery, and gestational diabetes mellitus (GDM). Laboratory parameters, all obtained from blood samples collected during the same admission prior to delivery, included urinary protein (24-h quantification), platelet count (PLT), D-dimer (D-D), fibrinogen (FIB), thrombin time (TT), prothrombin time (PT), activated partial thromboplastin time (APTT), uric acid (UA), serum creatinine (Cr), alanine aminotransferase (ALT), aspartate aminotransferase (AST), lactate dehydrogenase (LDH), hemoglobin (HGB), and placental growth factor (PlGF). Neonatal outcomes, including birth weight and birth length, were collected for descriptive analysis but were not used as input variables in the predictive modeling process. All variables were extracted from electronic medical records, and laboratory tests were conducted in the hospital’s central laboratory following standardized procedures.

### 2.4 Machine learning modeling and model interpretation

Before model development, data preprocessing was performed to ensure analytical transparency and reproducibility. Missing data imputation was not required because cases with incomplete demographic or laboratory information had been excluded during cohort selection. All continuous variables were standardized using z-score normalization, and categorical variables were encoded using one-hot encoding to ensure model compatibility. Given the moderate class imbalance in the dataset (25.9% placental abruption vs. 74.1% non-abruption), class-weighting was applied for algorithms that support weighted loss functions [logistic regression, support vector machine (SVM), random forest (RF), extreme gradient boosting (XGBoost)] to prevent bias toward the majority class. Furthermore, to reduce the risk of model overestimation associated with a single 70/30 split, hyperparameter tuning for all ML models was conducted using five-fold cross-validation within the training dataset, while the validation set was reserved solely for final performance assessment. A comprehensive ML workflow was designed to develop and validate predictive models for placental abruption. The process consisted of four major steps: feature selection, model construction, performance evaluation, and model interpretation.

#### 2.4.1 Feature selection

To reduce data dimensionality and avoid overfitting, we implemented a dual-step feature selection strategy. First, the least absolute shrinkage and selection operator (LASSO) regression was applied to identify predictors with nonzero coefficients by introducing an L1 regularization penalty, which effectively eliminates redundant or weakly informative variables. Second, the Boruta algorithm, a wrapper method built upon the random forest classifier, was used to confirm the relevance of each variable through iterative comparison with randomized shadow features. The intersection of the variables selected by both methods was used as the final feature set for model training. To evaluate the robustness of the selected predictors, both Boruta and LASSO procedures were embedded within the five-fold cross-validation framework used in model tuning. Predictor stability was assessed by examining the frequency with which each variable was selected across folds.

#### 2.4.2 Model construction

Six supervised learning algorithms were employed for model training: decision tree (DT), k-nearest neighbor (KNN), logistic regression, RF, SVM, and XGBoost. The dataset was randomly divided into training and validation subsets at a ratio of 7:3. The training set was used for model fitting, while the validation set was reserved for independent performance assessment. All continuous variables were normalized or standardized as appropriate, and categorical variables were encoded to ensure algorithm compatibility. Hyperparameters for each model were optimized through grid search combined with five-fold cross-validation on the training data to achieve optimal predictive performance. For tree-based algorithms (e.g., DT, RF, XGBoost), the hyperparameter search grid included depth-related constraints—such as maximum tree depth, minimum samples per split, and minimum samples per leaf—as well as parameters regulating the number of features considered at each split. These constraints were incorporated intentionally to limit model complexity and reduce high-variance behavior. All hyperparameters were selected via five-fold cross-validation within the training set.

#### 2.4.3 Performance evaluation

Model performance was comprehensively assessed using several quantitative indicators, including the area under the receiver operating characteristic (ROC) curve (AUC), F1 score, accuracy, and other relevant metrics. Among these, AUC was used as the primary criterion for discriminative ability, while F1 score provided a balanced measure of precision and recall, especially under conditions of class imbalance. The model that achieved the best overall performance across these indicators was selected as the final predictive model. Subsequently, model reliability and clinical applicability were further assessed through calibration curve analysis (to evaluate agreement between predicted and observed probabilities), decision curve analysis (DCA) (to quantify net clinical benefit across different threshold probabilities), and visualization of the confusion matrix (to assess classification consistency).

#### 2.4.4 Model interpretation

To enhance interpretability and explore the contribution of each predictor to the model’s decisions, SHAP analysis was performed on the optimal model. SHAP values were used to decompose each individual prediction into additive feature contributions, illustrating both the direction and magnitude of each variable’s effect on the model output. Global feature importance ranking and SHAP summary plots were generated to visualize global feature impact, while local explanation plots were used to provide case-level interpretability and align model reasoning with clinical understanding (24, 25).

### 2.5 Statistical analysis

Before model construction, data preprocessing and descriptive analyses were conducted to evaluate the distribution patterns and variability of all variables. The normality of continuous variables was tested using the Shapiro–Wilk test. Variables conforming to a normal distribution were expressed as mean ± standard deviation (SD) and compared between groups using the independent samples *t*-test. Non-normally distributed variables were summarized as median (interquartile range, IQR) and analyzed using the Mann–Whitney U test. Categorical variables were reported as counts and percentages, and intergroup differences were examined with the Chi-square test. A dual-step feature selection combining LASSO regression and Boruta algorithm was applied to identify the most robust and non-redundant predictors for model construction. Multicollinearity among predictors was further examined using the variance inflation factor (VIF), and variables with VIF > 5 were excluded to avoid instability in feature selection and model estimation.

R (version 4.3.1) was used for all statistical analyses. Feature selection was conducted using the “glmnet” and “Boruta” packages. ROC analysis and AUC (95% CI) were calculated using the “pROC” package. Six supervised learning algorithms were implemented within the “tidymodels” framework. Calibration curves were generated using the “rms” package. DCA was conducted with the “rmda” package to assess the net clinical benefit across a range of threshold probabilities. Visualization of model performance and data distribution was completed using “ggplot2.” For interpretability, SHAP values were computed using the “shapviz” and “fastshap” packages. Two-sided tests were applied, with *p* < 0.05 considered statistically significant.

## 3 Results

### 3.1 Baseline comparison between the training and validation datasets

Among all 580 patients with EOPE, placental abruption occurred in 150 cases (25.9%), while 430 patients (74.1%) showed no abruption. The overall mean maternal age was 29.7 ± 4.2 years, and 80 women (13.8%) were aged ≥35 years. When comparing the two datasets, the incidence of placental abruption was 25.1% in the training set and 27.6% in the validation set, with no significant difference (*p* = 0.535). Similarly, maternal age and the proportion of women aged ≥35 years did not differ significantly between groups (*p* > 0.05). Other demographic, clinical, and laboratory variables were also comparable between the training and validation sets (all *p* > 0.05). Overall, the two datasets were well balanced, ensuring the reliability of subsequent model construction and validation (Table 1).

**Table 1:** Baseline clinical characteristics of patients in the training and validation sets.

Variables	Total (*n* = 580)	Training set (*n* = 406)	Validation set (*n* = 174)	*χ* ^2^ */t/Z*	*P*
Placental abruption				0.385	0.535
No	430 (74.1%)	304 (74.9%)	126 (72.4%)		
Yes	150 (25.9%)	102 (25.1%)	48 (27.6%)		
Age (years)	29.72 ± 4.21	29.58 ± 4.36	30.08 ± 3.85	1.310	0.191
Age (years)				2.486	0.115
≥35	80 (13.8%)	50 (12.3%)	30 (17.2%)		
<35	500 (86.2%)	356 (87.7%)	144 (82.8%)		
Prepregnancy BMI (kg/m^2^)	21.86 ± 2.69	21.92 ± 2.79	21.71 ± 2.48	0.858	0.391
Postpregnancy BMI (kg/m^2^)	27.11 ± 3.52	27.21 ± 3.63	26.92 ± 3.27	0.908	0.364
SBP (mmHg)	151.79 ± 12.04	151.67 ± 11.89	152.04 ± 12.33	0.340	0.734
DBP (mmHg)	93.77 ± 11.08	93.72 ± 11.05	93.89 ± 11.12	0.170	0.866
Severity				0.034	0.853
Severe	230 (39.7%)	162 (39.9%)	68 (39.1%)		
Mild	350 (60.3%)	244 (60.1%)	106 (60.9%)		
Gestational age (weeks)	36.01 ± 3.03	35.97 ± 3.08	36.11 ± 2.92	0.509	0.611
Number of pregnancies				0.047	0.828
≥3	130 (22.4%)	92 (22.7%)	38 (21.8%)		
<3	450 (77.6%)	314 (77.3%)	136 (78.2%)		
Mode of delivery				0.227	0.634
Cesarean section	248 (42.8%)	171 (42.1%)	77 (44.3%)		
Vaginal delivery	332 (57.2%)	235 (57.9%)	97 (55.7%)		
GDM				0.181	0.671
No	502 (86.6%)	353 (86.9%)	149 (85.6%)		
Yes	78 (13.4%)	53 (13.1%)	25 (14.4%)		
Smoking during pregnancy				3.370	0.066
No	494 (85.2%)	353 (86.9%)	141 (81.0%)		
Yes	86 (14.8%)	53 (13.1%)	33 (19.0%)		
Neonatal length (cm)	50.15 ± 2.16	50.10 ± 2.06	50.24 ± 2.35	0.718	0.473
Neonate birth weight (kg)	3.10 ± 0.47	3.08 ± 0.45	3.13 ± 0.52	1.169	0.243
Urinary protein (g/24 h)	2.38 (1.00, 3.62)	2.35 (0.80, 3.80)	2.40 (1.41, 3.23)	0.478	0.633
PLT (×10^9^/L)	144 (92, 211)	135 (92, 211)	167 (91, 212)	0.228	0.819
D-D (mg/L)	2.17 (1.10, 3.46)	2.16 (1.16, 3.41)	2.19 (0.91, 3.53)	0.208	0.835
FIB (g/L)	3.62 ± 1.08	3.61 ± 1.06	3.65 ± 1.11	0.411	0.682
TT (s)	14.61 ± 2.72	14.59 ± 2.79	14.64 ± 2.61	0.202	0.840
PT (s)	12.67 ± 3.04	12.64 ± 2.99	12.73 ± 3.14	0.327	0.744
APTT (s)	30.87 ± 4.03	30.92 ± 4.07	30.76 ± 3.95	0.438	0.662
UA (μmol/L)	372.11 ± 48.64	372.83 ± 47.01	370.63 ± 51.83	0.501	0.617
Cr (μmol/L)	77.3 (46.2, 104.5)	80.2 (47.0, 102.3)	68.0 (45.3, 107.8)	0.158	0.875
ALT (U/L)	20.37 ± 6.02	20.43 ± 6.20	20.26 ± 5.68	0.310	0.757
AST (U/L)	22.02 ± 5.16	21.97 ± 5.28	22.13 ± 4.98	0.340	0.734
LDH (U/L)	306.70 ± 52.90	307.46 ± 51.60	305.19 ± 55.37	0.475	0.635
HGB (g/L)	114.09 ± 21.65	114.65 ± 22.41	112.74 ± 19.88	0.972	0.331
PlGF (pg/mL)	99.16 ± 18.15	98.56 ± 18.85	100.53 ± 16.39	1.198	0.231

BMI, body mass index; SBP, systolic blood pressure; DBP, diastolic blood pressure; GDM, gestational diabetes mellitus; PLT, platelet count; D-D, D-dimer; FIB, fibrinogen; TT, thrombin time; PT, prothrombin time; APTT, activated partial thromboplastin time; UA, uric acid; Cr, creatinine; ALT, alanine aminotransferase; AST, aspartate aminotransferase; LDH, lactate dehydrogenase; HGB, hemoglobin; PlGF, placental growth factor.

### 3.2 Comparison between abruption and non-abruption groups in the training set

As shown in Table 2, patients with placental abruption were significantly older than those without. The proportion of women aged ≥ 35 years was also markedly higher in the abruption group (*p* < 0.001). Compared with patients without abruption, those who experienced placental abruption had a lower prepregnancy BMI, but no significant difference was observed in postpregnancy BMI. In terms of blood pressure, both systolic and diastolic levels were higher in the abruption group (*p* < 0.001). The proportion of severe PE was also significantly greater among those with placental abruption. Regarding behavioral factors, smoking during pregnancy was more frequent in the abruption group (*p* = 0.001). Among laboratory parameters, patients with placental abruption had significantly higher 24-h urinary protein level, but lower FIB and PlGF (all *p* < 0.001).

**Table 2:** Comparison of clinical and laboratory characteristics between patients with and without placental abruption in the training set.

Variables	Placental abruption (*n* = 102)	No-placental abruption (*n* = 304)	*χ* ^2^ */t/Z*	*P*
Age (years)	32.47 ± 4.27	28.62 ± 3.94	8.359	<0.001
Age (years)			32.765	<0.001
≥35	29 (28.4%)	21 (6.9%)		
<35	73 (71.6%)	283 (93.1%)		
Prepregnancy BMI (kg/m^2^)	20.78 ± 2.19	22.31 ± 2.87	4.923	<0.001
Postpregnancy BMI (kg/m^2^)	26.92 ± 3.22	27.31 ± 3.76	0.938	0.349
SBP (mmHg)	153.76 ± 13.27	150.97 ± 11.33	2.059	0.040
DBP (mmHg)	99.32 ± 10.51	91.84 ± 10.59	6.184	<0.001
Severity			24.773	<0.001
Severe	62 (60.8%)	100 (32.9%)		
Mild	40 (39.2%)	204 (67.1%)		
Gestational age (weeks)	35.66 ± 2.98	36.07 ± 3.11	1.164	0.245
Number of pregnancies			0.623	0.430
≥3	26 (25.5%)	66 (21.7%)		
<3	76 (74.5%)	238 (78.3%)		
Mode of delivery			0.001	0.993
Cesarean section	43 (42.2%)	128 (42.1%)		
Vaginal delivery	59 (57.8%)	176 (57.9%)		
GDM			0.200	0.655
No	90 (88.2%)	263 (86.5%)		
Yes	12 (11.8%)	41 (13.5%)		
Smoking during pregnancy			10.820	0.001
No	79 (77.5%)	274 (90.1%)		
Yes	23 (22.5%)	30 (9.9%)		
Neonatal length (cm)	49.78 ± 2.17	50.21 ± 2.01	1.832	0.068
Neonate birth weight (kg)	3.01 ± 0.39	3.10 ± 0.47	1.743	0.082
Urinary protein (g/24 h)	3.10 (1.99, 5.53)	1.41 (0.53, 3.40)	6.268	<0.001
PLT (×10^9^/L)	147 (91, 200)	135 (92, 215)	0.879	0.380
D-D (mg/L)	2.41 (1.12, 3.33)	2.15 (1.18, 3.45)	0.404	0.686
FIB (g/L)	3.14 ± 0.93	3.78 ± 1.05	5.476	<0.001
TT (s)	14.27 ± 2.46	14.69 ± 2.88	1.320	0.188
PT (s)	12.85 ± 2.71	12.56 ± 3.07	0.849	0.396
APTT (s)	30.59 ± 3.89	31.04 ± 4.13	0.966	0.335
UA (μmol/L)	368.77 ± 50.38	374.19 ± 45.82	1.008	0.314
Cr (μmol/L)	74.0 (57.5, 102.9)	81.4 (45.0, 102.7)	0.640	0.522
ALT (U/L)	19.46 ± 5.78	20.75 ± 6.31	1.824	0.069
AST (U/L)	22.56 ± 4.93	21.77 ± 5.39	1.308	0.192
LDH (U/L)	305.21 ± 46.72	308.22 ± 53.19	0.509	0.611
HGB (g/L)	112.67 ± 24.78	115.32 ± 21.56	1.034	0.302
PlGF (pg/mL)	90.26 ± 16.89	101.34 ± 18.67	5.308	<0.001

BMI, body mass index; SBP, systolic blood pressure; DBP, diastolic blood pressure; GDM, gestational diabetes mellitus; PLT, platelet count; D-D, D-dimer; FIB, fibrinogen; TT, thrombin time; PT, prothrombin time; APTT, activated partial thromboplastin time; UA, uric acid; Cr, creatinine; ALT, alanine aminotransferase; AST, aspartate aminotransferase; LDH, lactate dehydrogenase; HGB, hemoglobin; PlGF, placental growth factor.

### 3.3 Feature selection based on Boruta and LASSO algorithms

**Figure 2:**
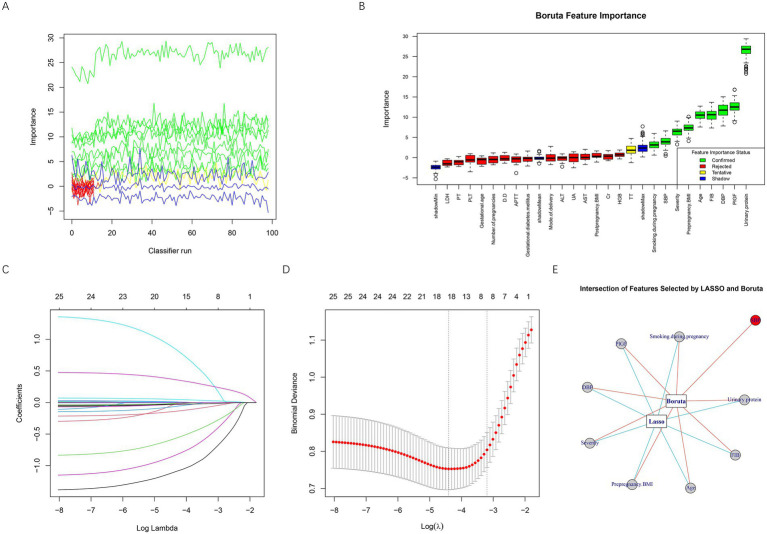
Feature selection based on Boruta and LASSO algorithms. **(A)** Iterative feature importance ranking generated by the Boruta algorithm during multiple classifier runs. **(B)** Boxplot of Boruta feature importance scores, showing confirmed important features in green, tentative features in yellow, rejected unimportant features in red, and shadow reference features in blue. **(C)** LASSO coefficient profiles for all variables plotted against log(λ). **(D)** Ten-fold cross-validation curve used to determine the optimal λ value in LASSO regression. The left dotted line represents the λ with the minimum binomial deviance (λ_min), while the right dotted line indicates the most regularized model within one standard error of the minimum (λ_1se). **(E)** Intersection of predictors identified by both Boruta and LASSO algorithms. BMI, body mass index; SBP, systolic blood pressure; DBP, diastolic blood pressure; GDM, gestational diabetes mellitus; PLT, platelet count; D-D, D-dimer; FIB, fibrinogen; TT, thrombin time; PT, prothrombin time; APTT, activated partial thromboplastin time; UA, uric acid; Cr, creatinine; ALT, alanine aminotransferase; AST, aspartate aminotransferase; LDH, lactate dehydrogenase; HGB, hemoglobin; PlGF, placental growth factor.

Feature selection was performed using both Boruta and LASSO algorithms to identify the most important predictors of placental abruption in patients with EOPE. As shown in Figures 2A,B, the Boruta algorithm iteratively assessed variable importance, confirming several key predictors (green boxplots) while rejecting irrelevant ones (red). The most influential features identified by Boruta included urinary protein, PlGF, DBP, age, FIB, prepregnancy BMI, severity, SBP, and smoking during pregnancy. In parallel, LASSO regression analysis (Figures 2C,D) was conducted to further reduce redundancy and prevent overfitting. The optimal regularization parameter (λ) was determined via 10-fold cross-validation, corresponding to the minimum binomial deviance. LASSO identified eight significant predictors—age, severity, FIB, smoking during pregnancy, urinary protein, prepregnancy BMI, DBP, and PlGF. Finally, the intersection of features selected by both Boruta and LASSO was visualized (Figure 2E), yielding eight robust predictors: urinary protein, PlGF, DBP, age, FIB, prepregnancy BMI, severity, and smoking during pregnancy. All eight final predictors appeared consistently in ≥80% of resampling iterations, indicating satisfactory stability and minimizing the risk of single-run bias. These variables were subsequently incorporated into model development for placental abruption prediction.

To examine potential collinearity among predictors in the model, VIFs were calculated before one-hot encoding of categorical variables, using the original variable structure, to preserve interpretability. All predictors demonstrated VIF values well below the accepted threshold of 5—age (1.07), prepregnancy BMI (1.05), DBP (1.08), severity (1.04), smoking during pregnancy (1.01), urinary protein (1.06), FIB (1.03), and PlGF (1.04)—suggesting that inter-variable correlations were minimal and did not pose a risk of multicollinearity. Table 3 presents the coding scheme used for variable assignment in the model.

**Table 3:** Variable assignment method.

Variables	Meaning	Value assignment
X_1_	Age	≥35 = 1, <35 = 2
X_2_	Prepregnancy BMI	Continuous variable
X_3_	DBP	Continuous variable
X_4_	Severity	Severe = 1, Mild = 2
X_5_	Smoking during pregnancy	No = 1, Yes = 2
X_6_	Urinary protein	Continuous variable
X_7_	FIB	Continuous variable
X_8_	PlGF	Continuous variable
Y	Placental abruption status	No-placental abruption = 0, Placental abruption = 1

BMI, body mass index; DBP, diastolic blood pressure; FIB, fibrinogen; PlGF, placental growth factor.

### 3.4 Model performance comparison and optimal model selection

**Figure 3:**
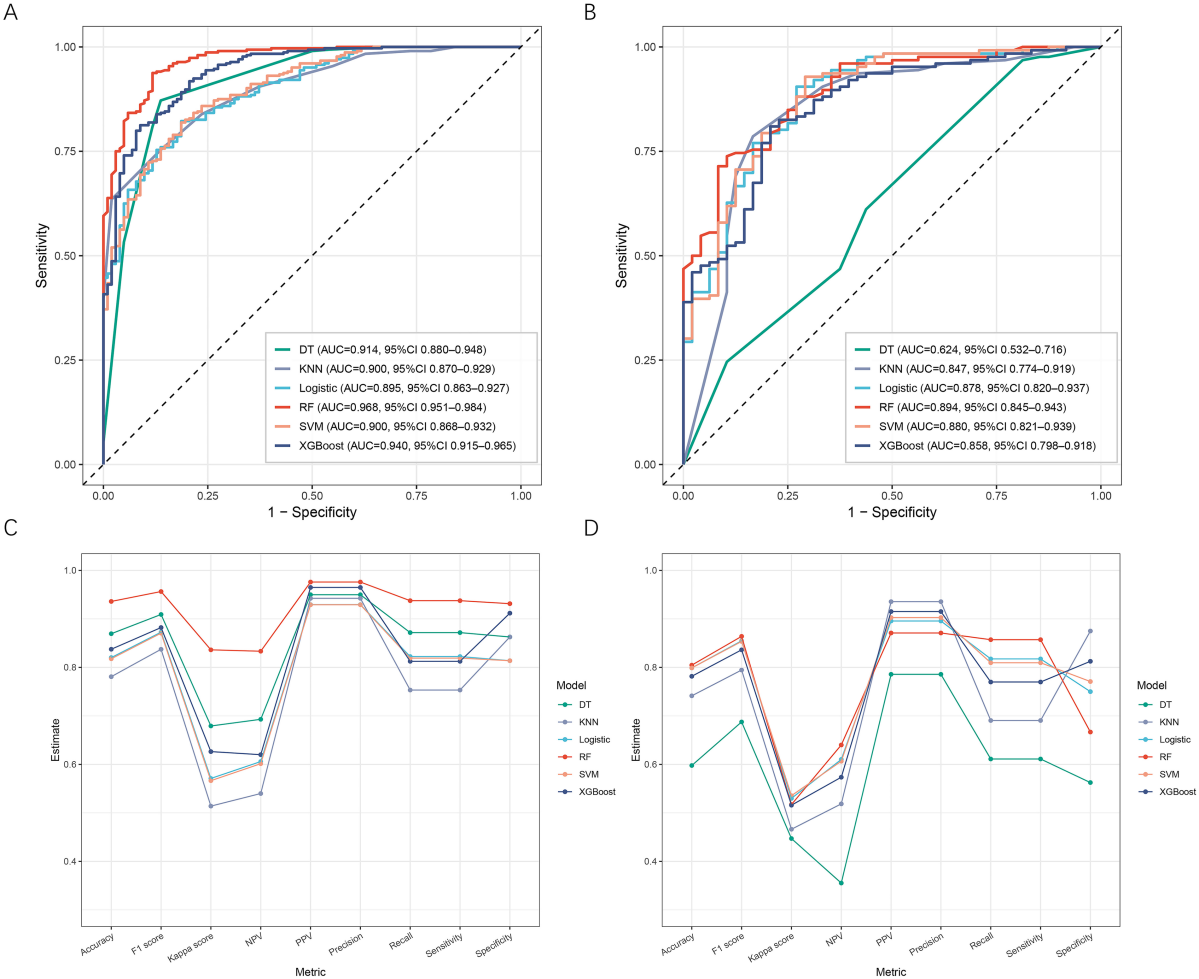
Comparison of model performance across six machine learning algorithms. **(A)** ROC curves of six models in the training set. **(B)** ROC curves of six models in the validation set. **(C)** Performance metrics of each model in the training set, including accuracy, F1 score, kappa, NPV, PPV, precision, recall, sensitivity, and specificity. **(D)** Corresponding performance metrics in the validation set. DT, decision tree; KNN, k-nearest neighbor; RF, random forest; SVM, support vector machine; XGBoost, extreme gradient boosting; NPV, negative predictive value; PPV, positive predictive value.

Six supervised machine learning algorithms, including DT, KNN, logistic regression, RF, SVM, and XGBoost, were developed to predict placental abruption in patients with EOPE. As shown in Figures 3A,B, the ROC curves demonstrated that all models exhibited favorable discriminative ability in both the training and validation datasets, with AUCs exceeding 0.80 except for DT in the validation cohort. Among them, RF achieved the highest AUC (training set: 0.968, 95% CI 0.951–0.984; validation set: 0.894, 95% CI 0.845–0.943), followed by SVM (AUC = 0.900 and 0.880, respectively). As illustrated in Figures 3C,D, the RF model outperformed the other algorithms across multiple performance metrics, including accuracy, F1 score, Cohen’s kappa, negative predictive value (NPV), recall, and sensitivity, in both the training and validation sets, indicating superior overall robustness and generalization ability. Taken together, the RF model was identified as the optimal predictive model for placental abruption and was subsequently used for interpretability analysis and clinical evaluation.

### 3.5 Validation and clinical applicability of the RF model

**Figure 4:**
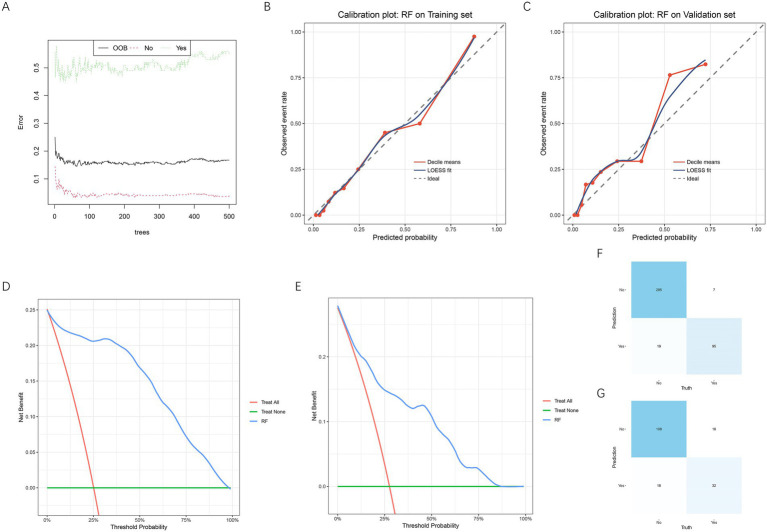
Evaluation of the performance and clinical applicability of the RF model. **(A)** Out-of-bag (OOB) error plot showing the convergence and stability of the RF model as the number of trees increases. The black line represents the overall OOB error rate, the red dotted line represents the error rate for the non-abruption group, and the green dotted line represents the error rate for the abruption group. **(B)** Calibration curve for the RF model in the training set. **(C)** Calibration curve for the RF model in the validation set. **(D)** DCA in the training set. **(E)** DCA in the validation set. **(F)** Confusion matrices for the training set. **(G)** Confusion matrices for the validation set. RF, random forest; OOB, out-of-bag; LOESS, locally estimated scatterplot smoothing.

The performance of the RF model was further evaluated for stability, calibration, discrimination, and clinical usefulness. As shown in Figure 4A, the out-of-bag (OOB) error rate gradually stabilized with the increasing number of decision trees, indicating good model convergence and internal consistency. The calibration plots demonstrated excellent agreement between the predicted and observed probabilities of placental abruption in both the training set (Figure 4B) and the validation set (Figure 4C), with the LOESS-fitted curve closely following the ideal diagonal line, suggesting good calibration. Importantly, the stability of these findings was supported by the five-fold cross-validation performed during hyperparameter tuning, which provided consistent performance estimates across resampled training subsets, reducing the risk of optimistic bias associated with a single 70/30 split. The DCA further indicated that the RF model provided a higher net clinical benefit than either the “treat all” or “treat none” strategies across a wide range of threshold probabilities in both datasets (Figures 4D,E). In addition, the confusion matrices (Figures 4F,G) showed that the RF model correctly classified the majority of patients, with high sensitivity and specificity in both the training and validation cohorts. These findings collectively confirm that the RF model exhibited excellent discrimination, calibration, and clinical utility for predicting placental abruption in early-onset preeclampsia.

### 3.6 Interpretation of the RF model using SHAP analysis

**Figure 5:**
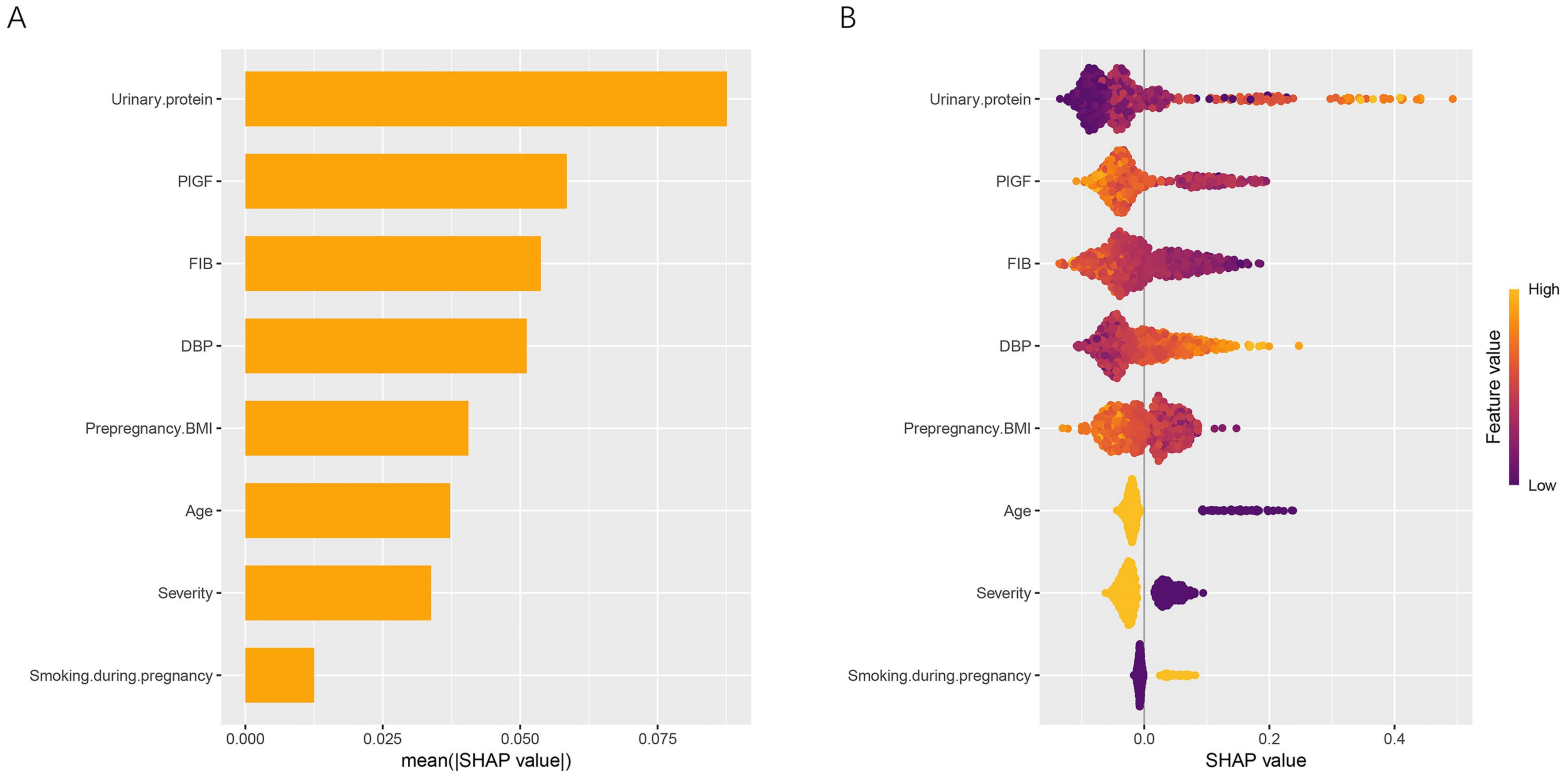
Global interpretation of the RF model using SHAP analysis. **(A)** Mean absolute SHAP values of eight predictors, illustrating their relative importance in the RF model. **(B)** SHAP summary plot showing both the magnitude and direction of each feature’s effect on the prediction. Feature values are color-coded from low (purple) to high (yellow). SHAP, SHapley Additive exPlanations; DBP, diastolic blood pressure; FIB, fibrinogen; PlGF, placental growth factor; BMI, body mass index.

**Figure 6:**
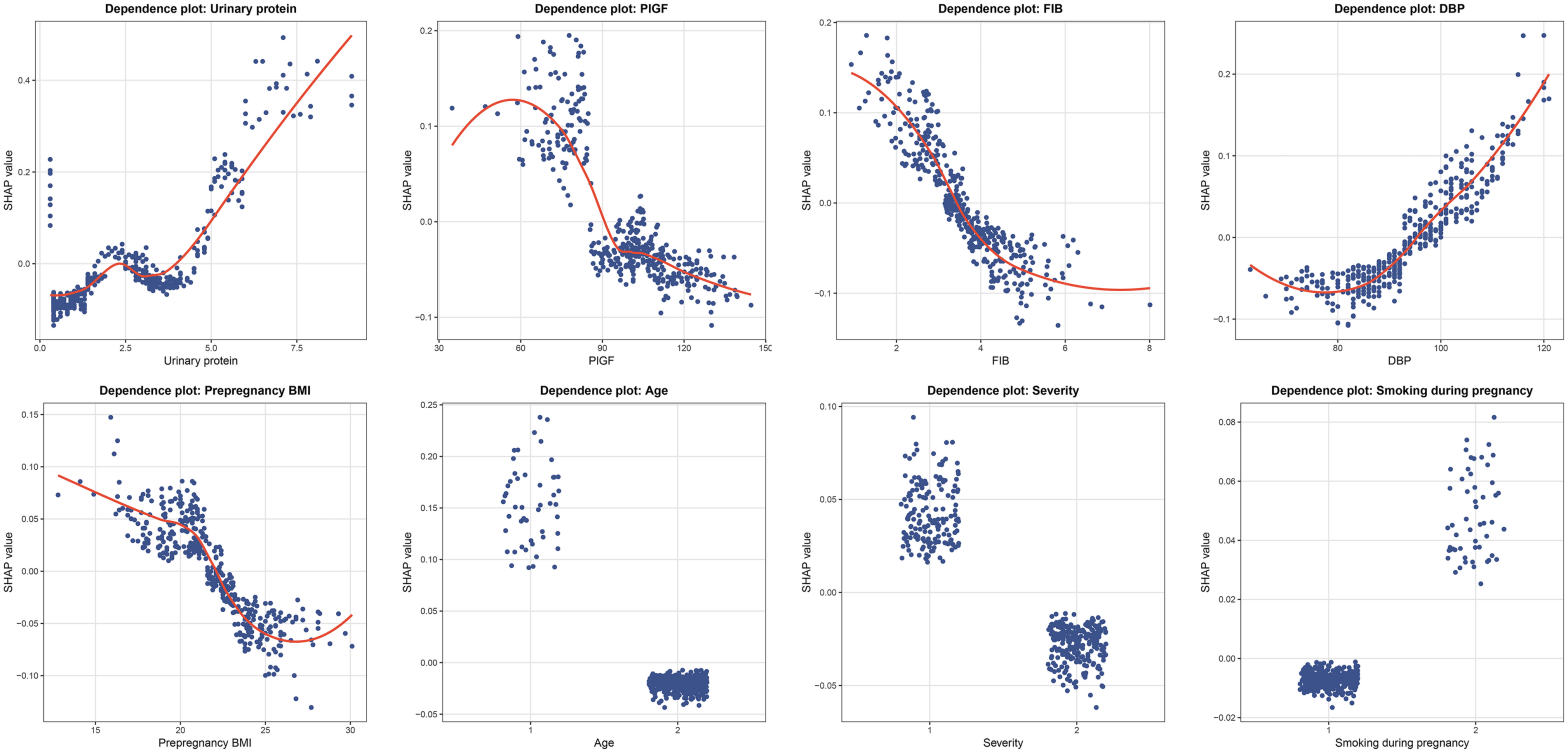
SHAP dependence plots of key predictors in the RF model. Each panel illustrates the nonlinear relationship between individual feature values and their corresponding SHAP values. Each dot represents one patient, where the x-axis shows the actual value of the variable and the y-axis indicates its SHAP value. DBP, diastolic blood pressure; FIB, fibrinogen; PlGF, placental growth factor; BMI, body mass index.

To elucidate the contribution and directional impact of each feature in the RF model, SHAP analysis was performed. As shown in Figure 5A, the mean absolute SHAP values indicated that urinary protein, PlGF, FIB, and DBP were the four most influential predictors, followed by prepregnancy BMI, age, severity, and smoking during pregnancy. Figure 5B presents the SHAP summary plot, which visualizes both the magnitude and direction of each feature’s effect on the prediction. Higher urinary protein and DBP levels were associated with an increased predicted risk of placental abruption, while higher FIB, PlGF, and prepregnancy BMI values contributed to a lower predicted risk. For categorical variables, patients aged ≥35 years (coded as 1) and those with severe preeclampsia (coded as 1) showed a higher predicted risk than their counterparts (<35 years or mild cases, coded as 2). In contrast, smoking during pregnancy (coded as 2) was associated with a higher SHAP value, suggesting that smokers had an elevated risk compared with nonsmokers. To further characterize these relationships, SHAP dependence plots were generated for all eight predictors (Figure 6). Urinary protein and DBP demonstrated a positive nonlinear association with SHAP values, suggesting that risk increased markedly beyond specific thresholds. Conversely, PlGF, FIB, and prepregnancy BMI exhibited negative trends, where higher values corresponded to lower predicted risk.

**Figure 7:**
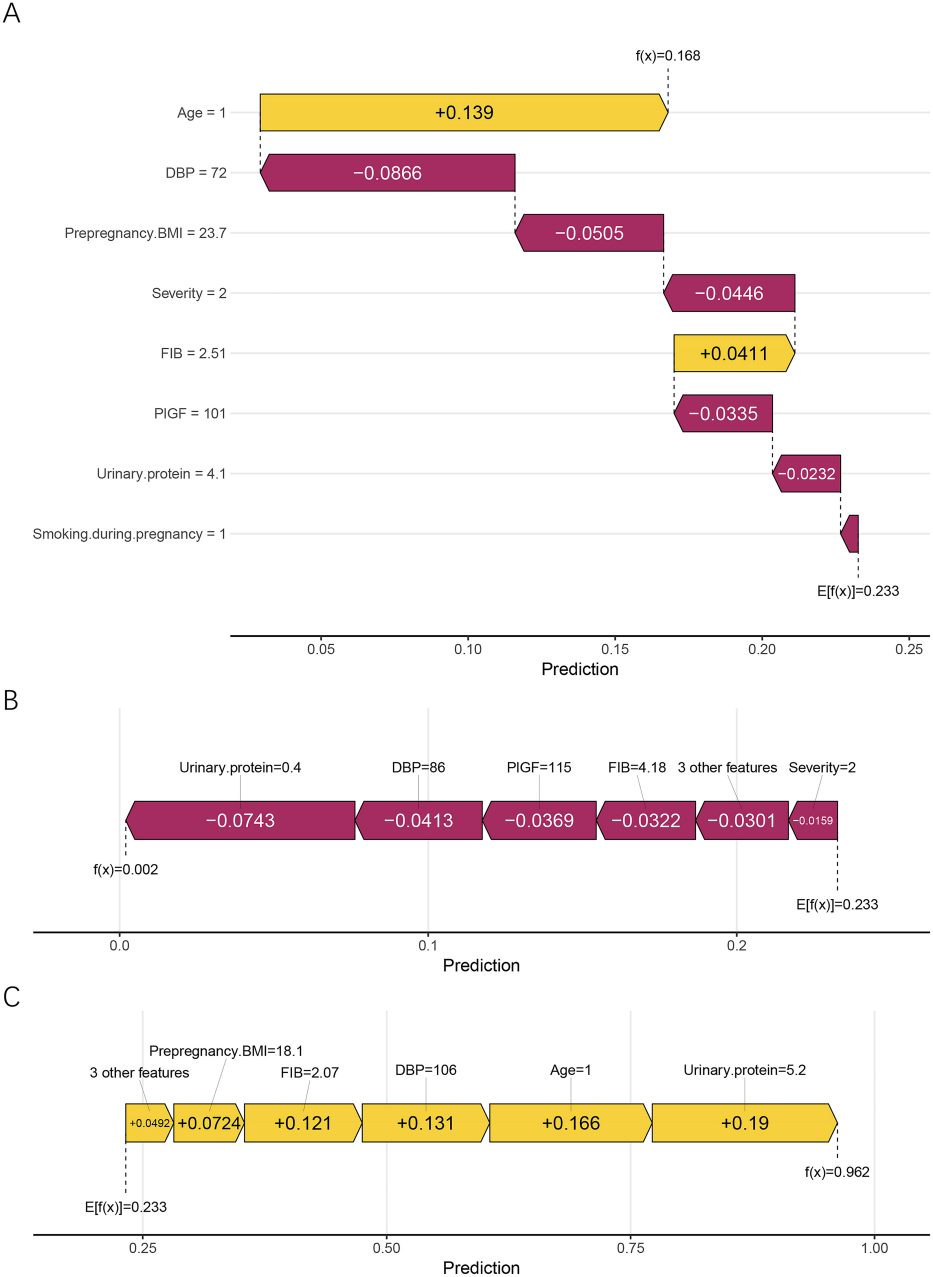
Local interpretation of the RF model using SHAP waterfall and force plots. **(A)** Corresponds to the first sample, demonstrating a balanced combination of risk-enhancing and protective factors. **(B)** Shows a low-risk case, in which low urinary protein, DBP, and high FIB jointly reduced the predicted probability. **(C)** Represents a high-risk case, characterized by elevated urinary protein and DBP, advanced maternal age (≥35 years), and low FIB, all of which positively contributed to the model output. Yellow bars indicate positive contributions (higher risk), whereas purple bars indicate negative contributions (lower risk). E[f(x)] represents the model’s baseline expected prediction, and f(x) denotes the individualized predicted probability. DBP, diastolic blood pressure; FIB, fibrinogen; PlGF, placental growth factor; BMI, body mass index.

In addition, local interpretability analysis was performed using SHAP waterfall and force plots to visualize the feature contributions for individual patients (Figure 7). Figure 7A presents the explanation for the first sample, whose predicted probability of placental abruption was moderate. In this case, the model attributes the increased risk primarily to age ≥35 years, while lower diastolic blood pressure (DBP) and higher prepregnancy BMI reduce the overall predicted value. Figure 7B shows a patient correctly classified as low-risk, in whom lower urinary protein and DBP, together with higher PlGF levels, collectively suppressed the predicted probability of abruption. In contrast, Figure 7C displays a high-risk case in which elevated urinary protein and DBP, advanced maternal age, and lower FIB levels jointly contributed to a strong positive prediction. Collectively, these SHAP analyses reveal both the global and individual interpretability of the RF model, highlighting how maternal, biochemical, and hemodynamic factors jointly shape the model’s risk estimation for placental abruption.

## 4 Discussion

In this study, we developed and validated an IML model to predict placental abruption among women with EOPE. Through a dual-step feature selection combining LASSO regression and the Boruta algorithm, eight key predictors were identified: urinary protein, PlGF, DBP, age, FIB, prepregnancy BMI, severity, and smoking during pregnancy. Among six supervised algorithms, the RF model achieved the best overall performance, with an AUC of 0.968 in the training set and 0.894 in the validation set. The RF model also demonstrated excellent calibration, robustness, and clinical utility, as evidenced by calibration curves and DCA. SHAP-based interpretation revealed that urinary protein, PlGF, FIB, and DBP were the dominant contributors to the prediction, providing both global and individual-level interpretability. Together, these findings indicate that an interpretable RF model can serve as a clinically valuable tool for early risk assessment and individualized management of placental abruption in EOPE.

Previous studies have attempted to develop predictive models for placental abruption or related complications in preeclampsia using mainly conventional statistical approaches. A study by Yang et al. established a validated risk-prediction model for placental abruption in PE patients, enrolling 1,448 cases with comprehensive clinical and biochemical data. Through multivariate logistic regression analysis, the authors identified several independent predictors, including PE severity, serum potassium level, DBP, and urinary casts, and subsequently constructed a nomogram that demonstrated good discrimination and calibration performance (26). Another retrospective study conducted in China included 303 cases of placental abruption and developed a “placental abruption prediction model” using multivariate logistic regression analysis. Independent risk factors identified in this model included advanced maternal age, multigravidity, hypertensive disorders complicating pregnancy, and anemia. The model demonstrated an AUC of 0.777, with a sensitivity of 67.3% and a specificity of 74.3% (27). Although these studies demonstrated the feasibility of risk prediction, most relied on linear modeling and lacked model transparency, limiting their ability to capture complex nonlinear interactions among biochemical, hemodynamic, and clinical factors. In contrast, our study applied a dual-step feature selection strategy combining LASSO regression and the Boruta algorithm to identify the most robust predictors, followed by the construction of multiple supervised learning models to enhance the model’s capacity to represent nonlinear and multivariable relationships. While previous models generally achieved AUC values approximately 0.8, the RF model in this study reached an AUC of 0.894 in the validation cohort, indicating a marked improvement in discriminative performance. Moreover, by integrating SHAP analysis, this study not only focused on predictive accuracy but also elucidated the direction and magnitude of each feature’s contribution to the outcome, providing transparent, clinically interpretable insights, whereas prior models largely remained at a purely predictive level with limited interpretability. In addition to conceptual advantages, the RF model demonstrated better empirical performance than a logistic regression baseline. Logistic regression achieved an AUC of 0.878 in the validation cohort, compared with 0.894 for the RF model, highlighting the superior ability of nonlinear ML methods to capture complex interactions among biochemical, hemodynamic, and clinical parameters in EOPE. This direct comparison reinforces the rationale for adopting ML-based approaches when traditional regression methods may underfit multifactorial obstetric conditions.

Building upon the model’s robust predictive capacity, further interpretation of the predictors provides insights into how maternal, hemodynamic, and biochemical factors jointly influence the development of placental abruption in EOPE. Advanced maternal age is an established risk factor for placental abruption. Women aged ≥35 years are more prone to vascular dysfunction and impaired uteroplacental perfusion, leading to placental ischemia and separation (9). A meta-analysis of 23 studies confirmed that advanced maternal age increases the risk of placental abruption (OR = 1.44, 95% CI 1.35–1.54), showing a consistent dose–response relationship (28). In our study, older age contributed positively to abruption risk, consistent with prior evidence linking maternal vascular aging to endothelial injury and decidual hypoxia. Low prepregnancy BMI may reflect poor maternal nutritional status and reduced placental reserve, leading to defective trophoblastic invasion and vulnerability to ischemia–reperfusion injury (29). A meta-analysis including 21 studies reported that women who were underweight before pregnancy had a moderately increased risk of placental abruption compared with those of normal weight (OR = 1.4, 95% CI 1.1–1.7), while overweight or obese women showed slightly lower risk (30). We also found that a lower pre-pregnancy BMI was associated with an increased risk of placental abruption, consistent with the theory that inadequate maternal metabolic adaptation may lead to impaired placental development in preeclampsia. Elevated DBP increases the risk of placental abruption by causing vasoconstriction and reduced uteroplacental perfusion. In PE, sustained DBP elevation worsens endothelial injury and microvascular damage (31, 32). Our study also confirmed DBP as a key predictor of placental abruption in EOPE. Severe PE markedly increases the risk of placental abruption due to profound endothelial dysfunction, excessive vasoconstriction, and abnormal placental perfusion. The degree of maternal hypertension and systemic involvement (such as hepatic or renal impairment) correlates with the extent of placental ischemia and decidual vascular injury, which can trigger premature placental separation (33, 34). Consistent with this pathophysiology, our cohort showed a higher abruption risk in severe PE compared with mild disease. Smoking during pregnancy is a well-established risk factor for placental abruption. A large Japanese cohort study found that both active smoking and secondhand smoke exposure during pregnancy significantly increased the risk of placental abruption, emphasizing that tobacco exposure, direct or indirect, substantially contributes to abruption risk (35). Cigarette smoke induces chronic hypoxia, oxidative stress, and vasoconstriction in the uteroplacental circulation, which compromise placental perfusion and promote decidual vascular injury (36, 37). In our cohort, smoking during pregnancy was also associated with a higher incidence of placental abruption, consistent with these previous findings. Proteinuria reflects renal endothelial injury and generalized microvascular dysfunction in preeclampsia. The resulting capillary leakage and intravascular volume depletion can further impair uteroplacental perfusion, predisposing to placental ischemia and abruption (38). A retrospective study of 150 preeclamptic pregnancies revealed that increasing proteinuria severity was closely associated with progressive maternal vascular malperfusion and earlier preterm delivery, suggesting that heavy proteinuria indicates more severe placental and endothelial damage (39). Consistent with these findings, our study identified urinary protein as the most influential predictor of placental abruption in EOPE, highlighting its central role in reflecting placental injury severity. Low FIB levels indicate consumptive coagulopathy and excessive fibrinolysis, which can aggravate decidual vascular injury and bleeding. Clinical studies have shown that hypofibrinogenemia is associated with severe maternal complications in placental abruption (40). Consistently, our results showed that lower FIB levels were linked to a higher risk of abruption in EOPE. Maternal circulating PlGF, an angiogenic factor predominantly released by the placenta, plays a key role in placental vascularization and perfusion (41). Lower PlGF levels have been linked to increased risk of placental vascular disorders, including placental abruption (42). In our cohort, decreased PlGF contributed significantly to the prediction of abruption, supporting the importance of angiogenic imbalance in EOPE-associated placental detachment.

By integrating LASSO regression and the Boruta algorithm, we identified the most robust and non-redundant predictors from a large set of clinical and laboratory variables. This dual-step screening approach ensured model parsimony, minimized multicollinearity, and retained features with the highest biological relevance to placental pathology. The apparent discrepancy between Boruta and LASSO in variable retention reflects their fundamentally different selection mechanisms. Boruta is designed to identify any variable with non-random predictive value, including correlated or nonlinear contributors, whereas LASSO emphasizes model sparsity through L1 penalization. As a result, variables such as SBP, although clinically meaningful and initially flagged by Boruta, were removed by LASSO because their information overlapped substantially with retained predictors such as D-D, FIB, and LDH. This complementary action of the two methods contributed to a stable and non-redundant final feature set. Subsequently, six supervised learning algorithms were compared, and the RF model demonstrated the best overall performance across AUC, F1 score, and calibration metrics, forming the optimal predictive framework. The IML framework applied in this study not only achieved high predictive accuracy but also provided transparent insights into how individual features contributed to the risk of placental abruption in EOPE. The visualization of SHAP summary and dependence plots allowed quantitative assessment of each variable’s directional effect, while local explanation plots enabled case-level interpretation, facilitating alignment between model reasoning and clinical intuition. From a clinical perspective, the integration of IML enhances trust and usability of AI-based decision support by allowing obstetricians to trace how risk predictions are derived for each patient. Compared with traditional “black-box” models, the SHAP-based RF framework offers both predictive power and interpretive transparency, supporting individualized counseling, early intervention, and more targeted monitoring strategies. To strengthen the clinical relevance of the model, our SHAP-based interpretation also provides actionable insights for patient management. High SHAP values for urinary protein or markedly reduced PlGF—identified as the strongest contributors in the model—may flag patients at imminent risk of placental abruption, suggesting the need for intensified maternal–fetal monitoring, earlier initiation of corticosteroids for fetal lung maturation, and closer evaluation for possible expedited delivery. Elevated SHAP contributions from DBP or low FIB can further inform clinical priorities, such as more aggressive blood pressure control, coagulation assessment, or preparedness for hemorrhagic complications. By translating feature contributions into individualized risk reasoning, the model supports real-time decision-making and enables clinicians to anticipate deterioration rather than react to it. However, several limitations should be acknowledged. First, although a relatively comprehensive set of clinical and laboratory variables was included, certain potentially important biomarkers, such as VEGF, uterine artery Doppler indices, oxidative stress markers, and placental imaging parameters, were unavailable for a proportion of patients, potentially underestimating the model’s biological interpretability. Second, this study focused primarily on EOPE-related placental abruption, and the model may not generalize to late-onset PE or normotensive abruption. Subgroup-specific models may be necessary to capture heterogeneity across clinical phenotypes. Third, despite the dual-step feature selection strategy and the stability supported by five-fold cross-validation, feature selection methods such as LASSO and Boruta may still exhibit variability across different samples. Future studies with larger and more diverse cohorts are needed to further evaluate the reproducibility of the selected predictors and the consistency of their contributions. Finally, the model was developed and validated within a single-center cohort without external validation or repeated resampling beyond cross-validation during model tuning. Therefore, the generalizability and robustness of the model require further confirmation in multicenter or prospective datasets.

## 5 Conclusion

This study developed and validated an IML model for predicting placental abruption in patients with EOPE. By integrating dual-step feature selection using LASSO regression and the Boruta algorithm, followed by comparative evaluation of six supervised learning algorithms, we identified the RF model as the optimal predictive framework with excellent discrimination and calibration. The SHAP-based interpretability framework further illustrated how key variables contributed to individualized risk predictions, providing transparent and clinically meaningful explanations. Our findings highlight the potential of IML to bridge predictive analytics and clinical reasoning. This transparent, data-driven approach enables individualized risk assessment and may facilitate early intervention and targeted surveillance for high-risk EOPE patients. Future multicenter, prospective studies are warranted to externally validate the model and explore its integration into routine obstetric care.

## Data Availability

The raw data supporting the conclusions of this article will be made available by the authors, without undue reservation.
